# Suppression of ERK phosphorylation through oxidative stress is involved in the mechanism underlying sevoflurane-induced toxicity in the developing brain

**DOI:** 10.1038/srep21859

**Published:** 2016-02-24

**Authors:** Shinya Yufune, Yasushi Satoh, Ryosuke Akai, Yosuke Yoshinaga, Yasushi Kobayashi, Shogo Endo, Tomiei Kazama

**Affiliations:** 1 Department of Anesthesiology, National Defense Medical College, 3-2 Namiki, Tokorozawa 359-8513, Japan; 2 Department of Obstetrics and Gynecology, Japan Self-Defense Forces Central Hospital, 1-2-24 Ikejiri, Setagaya, Tokyo 154-8532, Japan; 3 Department of Anatomy and Neurobiology, National Defense Medical College, 3-2 Namiki, Tokorozawa 359-8513, Japan; 4 Aging Neuroscience Research Team, Tokyo Metropolitan Geriatric Hospital and Institute of Gerontology, 35-2 Sakaecho, Itabashi, Tokyo 173-0015, Japan

## Abstract

In animal models, neonatal exposure to general anesthetics significantly increased neuronal apoptosis with subsequent behavioral deficits in adulthood. Although the underlying mechanism is largely unknown, involvement of extracellular signal-regulated kinases (ERKs) is speculated since ERK phosphorylation is decreased by neonatal anesthetic exposure. Importance of ERK phosphorylation for neuronal development is underscored by our recent finding that transient suppression of ERK phosphorylation during the neonatal period significantly increased neuronal apoptosis and induced behavioral deficits. However, it is still unknown as to what extent decreased ERK phosphorylation contributes to the mechanism underlying anesthetic-induced toxicity. Here we investigated the causal relationship of decreased ERK phosphorylation and anesthetic-induced toxicity in the developing brain. At postnatal day 6 (P6), mice were exposed to sevoflurane (2%) or the blood-brain barrier-penetrating MEK inhibitor, α-[amino[(4-aminophenyl)thio]methylene]-2-(trifluoromethyl)benzeneacetonitrile (SL327) (50 mg/kg). Transient suppression of ERK phosphorylation by an intraperitoneal injection of SL327 at P6 significantly increased apoptosis similar to sevoflurane-induced apoptosis. Conversely, SL327 administration at P14 or P21 did not induce apoptosis, even though ERK phosphorylation was inhibited. Restoring ERK phosphorylation by administration of molecular hydrogen ameliorated sevoflurane-induced apoptosis. Together, our results strongly suggests that suppressed ERK phosphorylation is critically involved in the mechanism underlying anesthetic-induced toxicity in the developing brain.

There is accumulating evidence indicating that several classes of anesthetics induce widespread apoptosis at clinically relevant concentration in the developing brain of a wide range of animals, including rodents^1^,^2^,^3^,^4^,^5^,^6^,^7^,^8^,^9^ and rhesus monkeys^10^,^11^. Furthermore, anesthetic administration causes long-term impairments later in adulthood^1^,^2^,^3^,^4^,^5^,^6^,^8^,^9^, raising concerns about the clinical use of general anesthetics in pediatric medicine. Although the underlying mechanism is largely unknown, it may involve ERK in its mechanism since ERK phosphorylation is decreased by neonatal anesthetic exposure^12^. ERKs are serine/threonine kinases that relay extracellular stimulation to intracellular responses and regulate a wide variety of cellular functions. Recently, we reported that transient suppression of ERK phosphorylation in mice by intraperitoneal injection of SL327 during the neonatal stage caused robust apoptosis in the brain, as well as had profound long-term effects on brain functioning, such as reduced long-term potentiation (LTP), impaired memory, and social deficits^13^. These results indicate that ERK phosphorylation have important roles in neuronal development during the neonatal stage. It should be noted that these effects caused by SL327 were very similar to those seen in mice exposed to general anesthetics during the neonatal period^3^. However, it is still unknown as to what extent and how decreased ERK phosphorylation contributes to the mechanism underlying anesthetic-induced toxicity in the developing brain.

As such, we hypothesized that reduced ERK phosphorylation may be critically involved in the mechanism underlying the apoptosis caused by neonatal exposure to general anesthetics. Thus, we examined how inhibition of ERK phosphorylation is associated with anesthetic-induced toxicity in the developing brain using a mouse model.

## Results

### Inhibition of ERK phosphorylation at P6 significantly increased apoptosis levels in the brain

**Figure 1:**
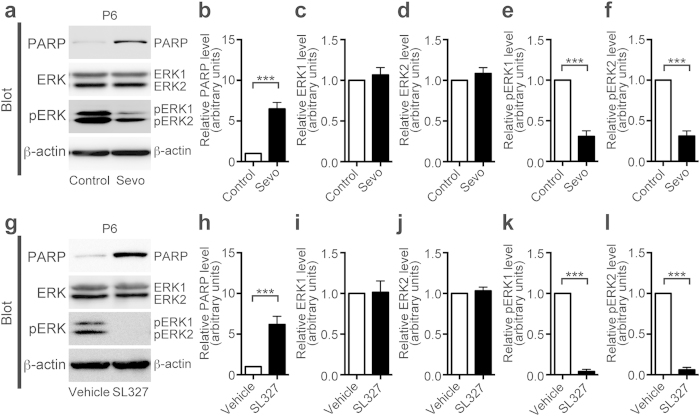
Pharmacological inhibition of ERK phosphorylation caused a significant increase in apoptosis at P6, similar to sevoflurane administration. (**a**) Representative western blot of forebrain protein extracts from mice treated with or without 2% sevoflurane for 6 h at P6 (sevoflurane (−) (control): n = 6; sevoflurane (+): n = 6). (**b**) Cleaved PARP levels significantly increased in sevoflurane-treated mice compared to the levels of cleaved PARP in controls. (**c,d**) Expression levels of ERK1 and ERK2 in sevoflurane-treated mice were not significantly affected compared to those of the controls. (**e,f**) ERK1 and ERK2 phosphorylation levels significantly decreased in sevoflurane-treated mice compared to those of the controls. (**g**) Representative western blot of forebrain protein extracts from mice treated with 50 mg/kg of SL327 or vehicle (DMSO) at P6 (vehicle: n = 6; SL327: n = 6). (**h**) Cleaved PARP levels significantly increased in SL327-treated mice compared to the levels of cleaved PARP in vehicle controls. (**i,j**) Expression levels of ERK1 and ERK2 were not significantly affected in SL327-treated mice compared to that in vehicle controls. (**k,l**) ERK1 and ERK2 phosphorylation levels significantly decreased in SL327-treated mice compared to their expression levels in vehicle controls. To evaluate the levels of protein expression and phosphorylation, band levels were divided according to their corresponding internal loading control (β-actin). Data are represented as mean ± SEM. ***P < 0.001.

To evaluate the effect of sevoflurane on ERK phosphorylation, we performed western blot analysis on forebrain tissue after 6 h-of anesthesia with 2% sevoflurane at P6 (Fig. 1a).

Sevoflurane-treated forebrain tissue had significantly increased levels of cleaved poly-(adenosine diphosphate-ribose) polymerase (PARP), compared to controls, indicating increased levels of apoptosis (Fig. 1b, t-test, t = 6.93, *P* < 0.0001). Sevoflurane exposure also significantly reduced the phosphorylation levels of both ERK isoforms (Fig. 1e,f: pERK1, t-test, t = 9.97, *P* < 0.0001; pERK2, t-test, t = 10.90, *P* < 0.0001). Total ERK expression levels were similar for both isoforms (Fig. 1c,d; t-test, *P* > 0.05).

Next, we examined the causal relationship of inhibition of ERK phosphorylation and apoptosis in the P6 mouse brain. We used SL327 to achieve transient suppression of ERK phosphorylation as our previous report^13^. SL327 can cross the blood-brain barrier and effectively reduce basal level of ERK phosphorylation in the CNS^14^,^15^. We previously reported that ERK phosphorylation levels reached a trough level above the concentration of 50 mg/kg SL327 6 h after SL327 administration^13^. Thus, in the current study, we investigated the effects of a single-dose intraperitoneal injection of SL327 at this concentration (Fig. 1g). SL327 significantly increased the levels of cleaved PARP compared to vehicle controls (Fig. 1h; t-test, t = 5.11, *P* = 0.0005), which is similar to our previous report^13^. This concentration of SL327 effectively blocked ERK phosphorylation (Fig. 1k,l: pERK1, t-test, t = 37.85, *P* < 0.0001; pERK2, t-test, t = 30.01, *P* < 0.0001) without decreasing total ERK expression levels for both isoforms (Fig. 1i,j; t-test, *P* > 0.05).

### Apoptosis caused by the inhibition of ERK phosphorylation is age-dependent

**Figure 2:**
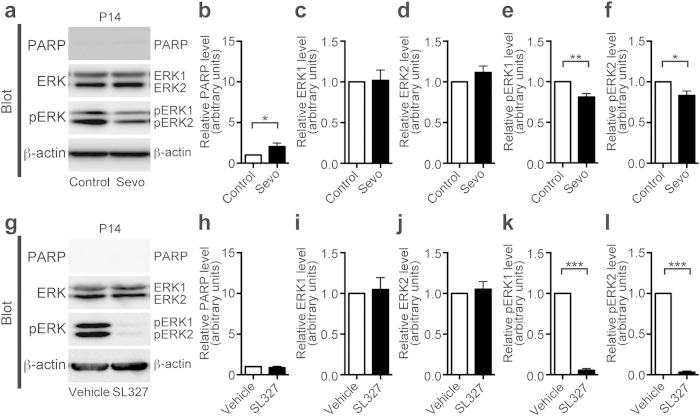
Inhibition of ERK phosphorylation by SL327 did not induce apoptosis at P14, although ERK phosphorylation levels significantly decreased. (**a**) Representative western blot of forebrain protein extracts from mice treated with or without 2% sevoflurane for 6 h at P14 (sevoflurane (−) (control): n = 6; sevoflurane (+): n = 6). (**b**) Cleaved PARP levels slightly increased in sevoflurane-treated mice compared to their levels in controls. (**c,d**) ERK1 and ERK2 expression levels in sevoflurane-treated mice were not significantly different compared to controls. (**e,f**) ERK1 and ERK2 phosphorylation levels slightly decreased in sevoflurane-treated mice compared to their levels in the controls. (**g**) Representative western blot of forebrain protein extracts from mice treated with 50 mg/kg SL327 or vehicle (DMSO) at P14 (vehicle: n = 6; SL327: n = 6). (**h**) Cleaved PARP levels were not significantly affected in SL327-treated mice compared to their expression levels in vehicle controls. (**i,j**) Expression levels of ERK1 and ERK2 in SL327-treated mice were not changed significantly compared with those of vehicle controls. (**k,l**) ERK1 and ERK2 phosphorylation levels significantly decreased in SL327-treated mice compared to their phosphorylation levels in vehicle controls. The protein expression and phosphorylation levels were calculated as described in Fig. 1. Data are represented as mean ± SEM. *P < 0.05, **P < 0.01, ***P < 0.001.

We also evaluated the effects of sevoflurane on ERK phosphorylation at P14 (Fig. 2a). Although sevoflurane significantly increased the levels of cleaved PARP compared to controls at P14 (Fig. 2b; t-test, t = 2.48, *P* = 0.035), this increase was relatively small compared to the increase seen at P6 (Fig. 1b). ERK phosphorylation levels decreased after sevoflurane treatment compared to the ERK phosphorylation levels of the controls (Fig. 2e,f: pERK1, t-test, t = 4.42, *P* = 0.0013; pERK2, t-test, t = 3.06, *P* = 0.012), although this reduction was relatively small compared to the reduction at P6 (Fig. 1e,f).

**Figure 3:**
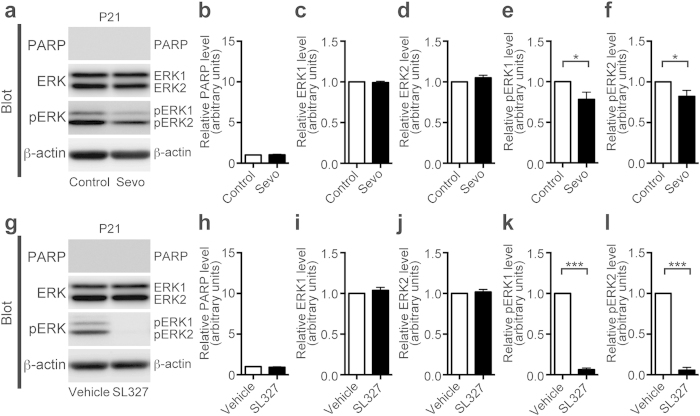
Neither sevoflurane nor SL327 induced apoptosis at P21, although ERK phosphorylation decreased. (**a**) Representative western blot of forebrain protein extracts from mice treated with or without 2% sevoflurane for 6 h at P21 (sevoflurane (−) (control): n = 5; sevoflurane (+): n = 5). (**b**) Cleaved PARP levels were not significantly affected in sevoflurane-treated mice compared to their levels in controls. (**c,d**) ERK1 and ERK2 expression levels in sevoflurane-treated mice were not significantly affected compared to their expression levels in controls. (**e,f**) ERK1 and ERK2 phosphorylation levels slightly decreased in sevoflurane-treated mice compared to their levels in controls. (**g**) Representative western blot of forebrain protein extracts from mice treated with 50 mg/kg SL327 or vehicle (DMSO) at P21 (vehicle control: n = 5; SL327: n = 5). (**h**) Cleaved PARP levels were not significantly affected in SL327-treated mice compared to their levels in vehicle controls. (**i,j**) ERK1 and ERK2 expression levels in SL327-treated mice were not significantly affected compared to their expression levels in vehicle controls. (**k,l**) ERK1 and ERK2 phosphorylation levels significantly decreased in SL327-treated mice compared to their levels in vehicle controls. The protein expression and phosphorylation levels were calculated as described in Fig. 1. Data are represented as mean ± SEM. *P < 0.05, ***P < 0.001.

The result prompted us to speculate that increase in apoptosis may be in proportion to the reduction in ERK phosphorylation. To test this hypothesis, we evaluated the relationship between apoptosis and ERK phosphorylation in mice administered SL327 (50 mg/kg) at P14 (Fig. 2g). Although ERK phosphorylation level was mostly (~95%) abolished by SL327 (Fig. 2k,l: pERK1, t-test, t = 43.61, *P* < 0.0001; pERK2, t-test, t = 66.21, *P* < 0.001), similar to the reduction at P6, apoptosis was not significantly altered by SL327 administration (Fig. 2h). These data indicate that our hypothesis was incorrect, and suggest that the P6 brain may be more susceptible to a reduction of ERK phosphorylation compared to the P14 brain. This hypothesis was confirmed by our findings in the P21 brain (Fig. 3a,g). Mice administered sevoflurane at P21 did not exhibit significantly altered cleaved PARP levels compared to controls (Fig. 3b; t-test, *P* > 0.05), although ERK phosphorylation levels were attenuated (Fig. 3e,f: pERK1, t-test, t = 2.47, *P* < 0.039; pERK2, t-test, t = 2.45, *P* = 0.04). Similarly, mice administered SL327 at P21 did not exhibit significantly altered cleaved PARP levels compared to vehicle controls (Fig. 3h; t-test, *P* > 0.05), even though ERK phosphorylation was mostly (~94%) abolished in the SL327 group (Fig. 3k,l: pERK1, t-test, t = 42.32, *P* < 0.0001; pERK2, t-test, t = 27.26, *P* < 0.0001). Thus, at P14 or P21, the reduction in ERK phosphorylation by SL327 did not significantly increase level of apoptosis. Taken together, we can conclude that apoptosis caused by SL327 is age-dependent, similar to the sevoflurane-induced apoptosis. In addition, the reduction of ERK phosphorylation by sevoflurane at P6 appears to be larger than the reduction at P14 and P21, suggesting that basal ERK phosphorylation may be more susceptible to sevoflurane exposure at P6 compared to P14 and P21.

### Transient suppression of ERK phosphorylation by SL327 induced apoptosis in a pattern similar to sevoflurane at P6

**Figure 4:**
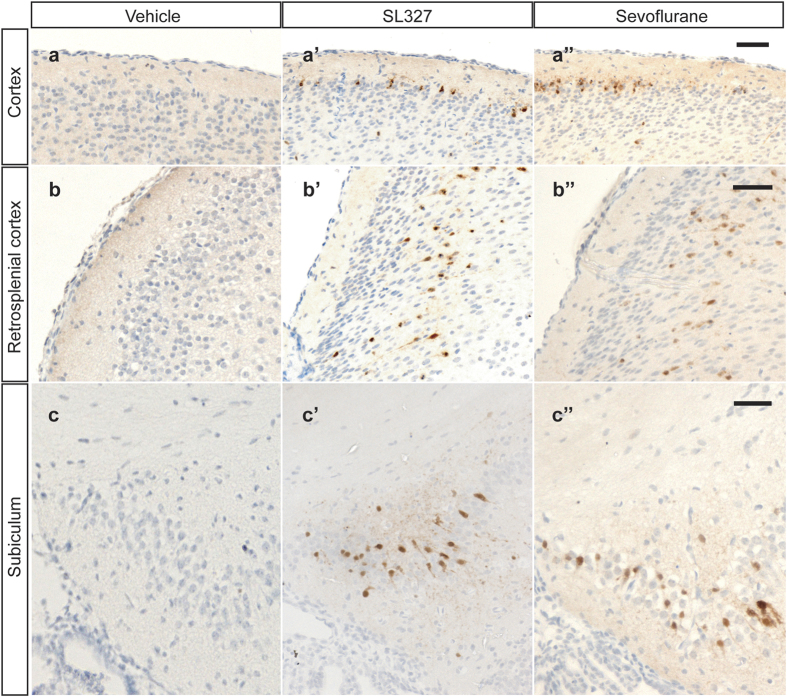
SL327-induced apoptosis had a similar anatomical pattern as sevoflurane-induced apopptosis at P6. Representative high-power images of the brains of mice treated with vehicle (**a**–**c**), sevoflurane (**a’**–**c**’), or SL327 (**a”**–**c”**). (**a,a’,a”**) cortex, (**b,b’,b”**) retrosplenial cortex, (**c,c’,c”**) subiculum. Scale bars: 250 μm.

Immunohistochemical analysis confirmed that the number of cells with activated (cleaved) caspase-3^+^ (AC3^+^) significantly increased in mice treated with SL327 at P6 compared to vehicle controls (Fig. 4), which is similar to that reported in our previous study^13^. In the current study, we confirmed that the localization of AC3+ immunoreactive cells in the SL327 group was similar to that of the sevoflurane group (Fig. 4).

**Figure 5:**
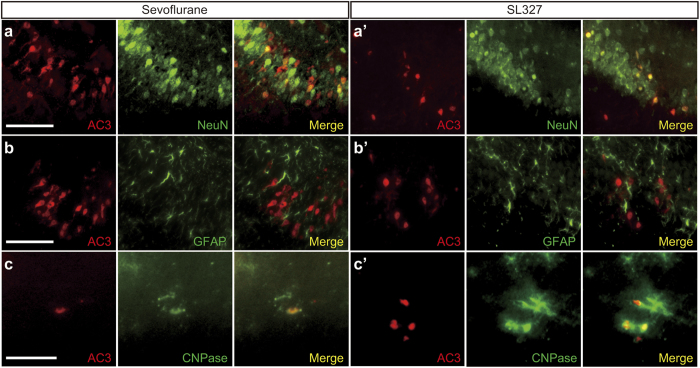
Cell types undergoing apoptosis were similar in mice administered SL327 and sevoflurane. Double immunostaining of activated caspase-3 with cellular markers for neurons (**a,a’**), astrocytes (**b,b’**), and oligodendrocytes (**c,c’**). (**a–c**) Merged images indicate that activated caspase-3 signals were observed in neurons (**a**) and oligodendrocytes (**c**), but not in astrocytes (**b**), 6 h after sevoflurane administration. (**a’–c’**) Similarly, activated caspase-3 signals were observed in neurons (**a’**) and oligodendrocytes (**c’**), but not in astrocytes (**b**), 6 h after SL327 injection. (**a,a’,b,b’**) dorsal hippocampal commissure; (**c,c’**) cortex. Scale bars: 50 μm.

Using cell type-specific markers, the subtypes of AC3^+^ cells were further examined. Double labeling for AC3 and the neuron-specific marker, neuronal nuclear antigen (NeuN), revealed that apoptosis was induced in neurons in the sevoflurane- (Fig. 5a) and SL327-treatment groups (Fig. 5a’). Conversely, the absence of double-labeled cell bodies for AC3 and the astrocyte-specific marker, glial fibrillary acidic protein (GFAP), indicated that apoptosis was not induced in astrocytes in sevoflurane- (Fig. 5b) or SL327-treatment groups (Fig. 5b’). Double labeling for AC3 and the oligodendrocyte-specific marker, 2′3-cyclic nucleotide 3′-phosphodiesterase (CNPase) indicated that apoptosis was induced in oligodendrocytes as well as neurons in the sevoflurane- (Fig. 5c) and SL327-treatment groups (Fig. 5c’). However, AC3 signals did not co-localize with CNPase in some regions (e.g., subiculum; data not shown). Collectively, the types of AC3^+^ cells undergoing apoptosis were similar in mice administered SL327 and sevoflurane.

Analysis of arterial blood before and 6 h after SL327 administration revealed no significant differences in pH, partial pressure of arterial oxygen (PaO_2_), or partial pressure of arterial carbon dioxide (PaCO_2_) between the various groups (Table 1). Thus, SL327 administration did not have significant adverse effects on pH, PaO_2_, and PaCO_2_, indicating that apoptosis caused by SL327 is not attributable to an indirect effect such as hypoxia.

### Decreased ERK activation is involved in the mechanism of increased apoptosis due to sevoflurane in the developing brain

**Figure 6:**
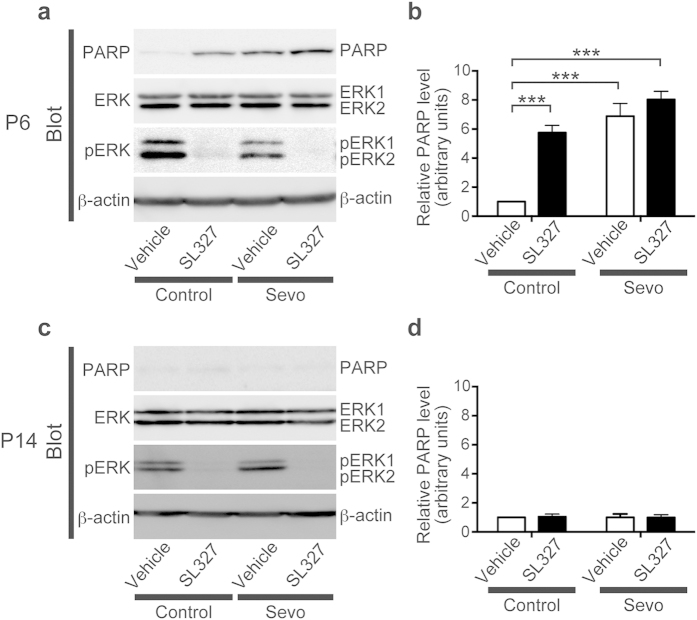
The involvement of the ERK pathway in sevoflurane-induced apoptosis at P6. (**a**) Representative western blot of forebrain protein extracts from mice treated with or without 2% sevoflurane in the presence of SL327 or vehicle at P6 (SL327 (−), sevoflurane (−): n = 8; SL327 (+), sevoflurane (−): n = 10; SL327 (−), sevoflurane (+): n = 8; SL327 (+), sevoflurane (+): n = 7). (**b**) SL327 administration at P6 significant increased cleaved PARP levels compared to vehicle controls when only carrier gas was administered (SL327 (−), sevoflurane (−) vs. SL327 (+), sevoflurane (−)). Conversely, no significant differences were observed between the SL327 and vehicle control groups when 2% sevoflurane was administered (SL327 (−), sevoflurane (+) vs. SL327 (+), sevoflurane (+)). (**c**) Representative western blot of forebrain protein extracts from mice treated with or without 2% sevoflurane in the presence of SL327 or vehicle at P14 (SL327 (−), sevoflurane (−): n = 6; SL327 (+), sevoflurane (−): n = 6; SL327 (−), sevoflurane (+): n = 6; SL327 (+), sevoflurane (+): n = 6). (**d**) No significant differences were observed in the cleaved PARP levels among the groups. To evaluate protein expression and phosphorylation, band levels were divided according to their corresponding internal loading control (β-actin). Data are represented as mean ± SEM. ***P < 0.001.

To further investigate the relationship between the ERK pathway and anesthetic-induced apoptosis, we evaluated the effect of SL327 administration on apoptosis in the brain during sevoflurane anesthesia. Mice at P6 were randomly assigned into one of the following groups: (1) SL327 (−), sevoflurane (−); (2) SL327 (+), sevoflurane (−); (3) SL327 (−), sevoflurane (+); or (4) SL327 (+), sevoflurane (+). Western blot analysis demonstrated that SL327 and/or sevoflurane administration induced a significant increase in apoptosis (Fig. 6a). A two-way ANOVA revealed a significant interaction between sevoflurane and SL327 treatments (*F* = 9.68, *P* = 0.0042), a significant main effect of sevoflurane treatment (*F* = 49.77, *P* < 0.0001), and a significant main effect of SL327 treatment (*F* = 25.92, *P* < 0.0001) (Fig. 6b). However, post hoc analysis indicated there were no significant differences in cleaved PARP levels between the (SL327 (−), sevoflurane (+)) group and the (SL327 (+), sevoflurane (+)) group (Fig. 6b; *P* > 0.05). Thus, the increased apoptosis caused by SL327 was occluded in mice exposed to sevoflurane. This support the idea that the decreased ERK phosphorylation is critically involved in the mechanism underlying anesthetic-induced toxicity in the developing brain.

### SL327 administration at P6 induced abnormal behaviors later in adulthood

**Figure 7:**
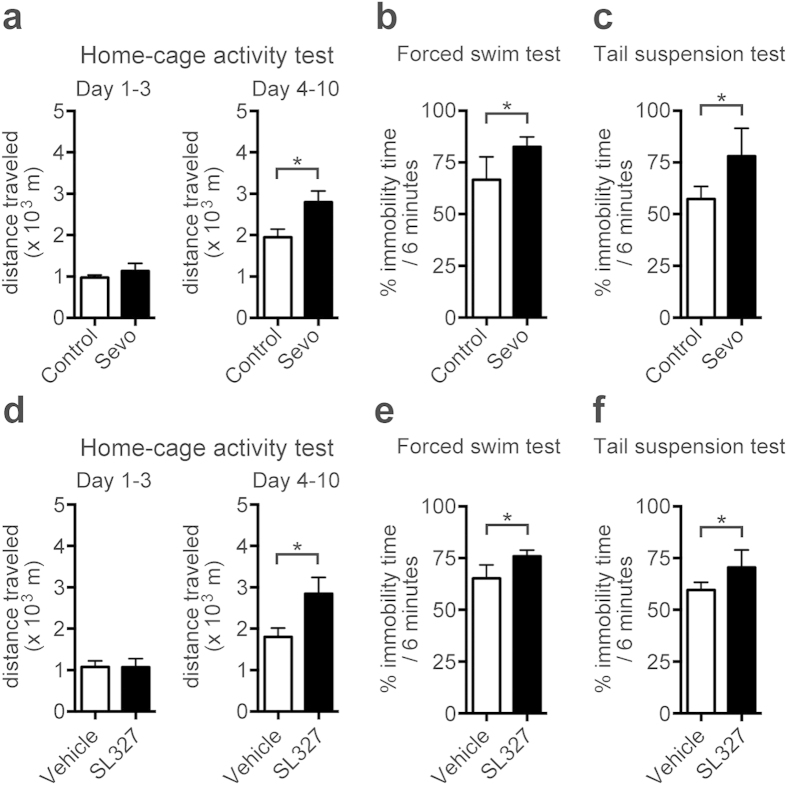
SL327 administration at P6 induced long-term deleterious effects, resulting in abnormal behaviors later in adulthood. (**a**) No significant differences were observed in total distance traveled over 3 consecutive days in a new cage at 10 weeks of age between mice treated with sevoflurane at P6 and controls (control: n = 7; sevoflurane: n = 7). Over an additional 7 days, the sevoflurane group travelled significantly longer distances compared to the vehicle group. (**b**) A significant difference was observed in immobility time in the forced swim test between mice treated with sevoflurane at P6 and controls (control: n = 7; sevoflurane: n = 7). (**c**) Significant difference was observed in immobility time in the tail suspension test between mice treated with sevoflurane at P6 and controls (control: n = 7; sevoflurane: n = 7). (**d**) No significant differences were observed in total distance traveled over 3 consecutive days in a new cage at 10 weeks of age between mice treated with SL327 at P6 and vehicle controls (control: n = 7; sevoflurane: n = 8). Over an additional 7 days, the sevoflurane group travelled significantly longer distances travelled compared to the vehicle group. (**e**) A significant difference was observed in the immobility time in the forced swim test between mice treated with SL327 at P6 and vehicle controls (control: n = 7; sevoflurane: n = 7). (**f**) A significant difference was observed in the immobility time in the tail suspension test between mice treated with SL327 at P6 and vehicle controls (control: n = 7; sevoflurane: n = 7). Data are represented as mean ± SEM. *P < 0.05.

We previously reported that SL327 administration at P6 induced learning impairments and social deficits later in adulthood^13^, similar to mice exposed to sevoflurane^3^. In the current study, we performed additional behavioral tests to examine the motor functions and depression like behavior in mice administered sevoflurane or SL327 at P6. First, mice were evaluated by the home-cage activity test to determine their motor activity. The sevoflurane group did not show any significant differences in the total distance travelled after placement in a new cage over the course of 3 consecutive days (12 hour light/12 hour dark cycle), compared to the control group (Fig. 7a; t-test, *P* > 0.05). In contrast, over an additional 7 days, the mice in the sevoflurane group travelled significantly longer distances compared to the mice in the control group (Fig. 7a; t-test, t = 2.52, *P* = 0.027). These data indicate that sevoflurane-treated mice are hyperactive in a familiar environment. Similarly, the total distance travelled by the mice in the SL327 group was not significantly different from that of the mice in the vehicle group (Fig. 7d; t-test, *P* > 0.05) over 3 consecutive days after placement in a new cage. However, SL327-treated mice travelled significantly longer distances over an additional 7 days, compared to the mice in the vehicle group (Fig. 7d; t-test, t = 2.23, *P* = 0.044). Therefore, SL327-treated mice also displayed a hyperactive phenotype in a familiar environment, similar to sevoflurane-treated mice.

Next, mice underwent the forced swim test, a widely used assay for assessing depressant-like behavior^16^. Mice administered sevoflurane at P6 exhibited a significant increase in immobility time (Fig. 7b; t-test, t = 2.50, *P* = 0.028), indicating increased depression-like behavior. Similarly, mice administered SL327 exhibited a significant increase in immobility time compared to vehicle controls (Fig. 7e; t-test, t = 2.75, *P* = 0.018). To further confirm the increased depression-like behavior in these mice, mice were also evaluated using the tail suspension test, another assay for assessing depression-like behavior^17^. Mice administered sevoflurane at P6 exhibited a significant increase in immobility time compared to controls (Fig. 7c; t-test, t = 2.61, *P* = 0.028), indicating increased depression-like behavior. Similarly, mice administered SL327 exhibited significantly increased immobility time compared to vehicle controls (Fig. 7f; t-test, t = 2.20, *P* = 0.049).

Together with our previous reports, these results indicate that SL327 administration at P6 induced pervasive behavioral deficits^13^. These deficits resemble those caused by sevoflurane exposure at the same neonatal time point^3^.

### Sevoflurane decreased ERK phosphorylation level via ROS accumulation

Although our data suggest that neonatal exposure to sevoflurane induced brain apoptosis through a decrease in ERK phosphorylation, the mechanism underlying the reduced ERK phosphorylation level by sevoflurane exposure remains unknown.

**Figure 8:**
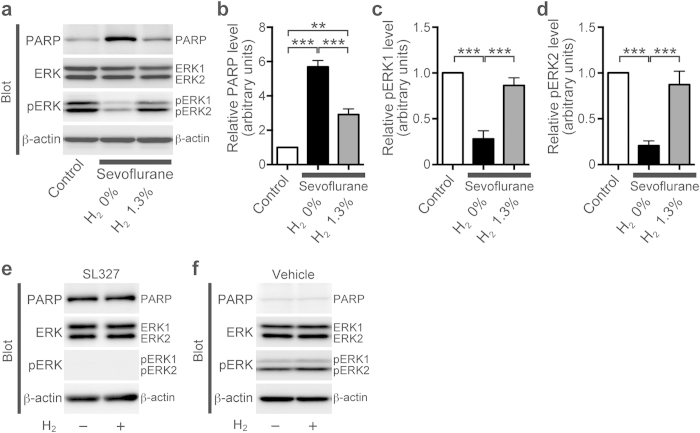
Sevoflurane decreased ERK phosphorylation through oxidative stress. (**a–d**) Molecular hydrogen (H_2_) gas, an effective antioxidant, decreased cleaved PARP levels caused by sevoflurane exposure at P6, concomitant with restored ERK phosphorylation levels. (**a**) Representative western blot of forebrain protein extracts from mice treated with or without 2% sevoflurane for 6 h in the presence of 1.3% hydrogen or not at P6 (sevoflurane (−), hydrogen (−): n = 5; sevoflurane (+), hydrogen (−): n = 5; sevoflurane (+), hydrogen (+): n = 5). (**b**) The increased levels of cleaved PARP after sevoflurane exposure were attenuated by hydrogen (1.3%) administration at P6. (**c,d**) The decreased ERK phosphorylation levels were restored by concomitant treatment with hydrogen (1.3%). (**e**) Representative western blot of forebrain protein extracts from mice treated with SL327 with or without hydrogen (SL327 (+), hydrogen (−): n = 5; SL327 (+), hydrogen (−): n = 6). (**f**) Representative western blot of forebrain protein extracts from mice treated with vehicle (DMSO) with or without hydrogen (SL327 (−), hydrogen (−): n = 5; SL327 (−), hydrogen (−): n = 5). To evaluate protein expression and phosphorylation, band levels were divided according to their corresponding internal loading control (β-actin). Data are represented as mean ± SEM. **P < 0.01, ***P < 0.001.

Accumulating evidence indicates that oxidative stress may contribute to anesthetic-induced neurotoxicity in the developing brain. It was reported that a synthetic ROS (reactive oxygen species) scavenger, EUK-134, effectively inhibited anesthetic-induced ROS production in the developing rat brain^18^. We also reported that molecular hydrogen (H_2_) gas, an effective antioxidant, suppressed anesthesia-induced ROS production in the developing mouse brain^19^. These antioxidants alleviated brain apoptosis caused by neonatal anesthetic exposure and subsequently alleviated behavioral deficits^18^,^19^. In the current study, we investigated the relationship between oxidative stress, reduced ERK phosphorylation, and the mechanism underlying anesthetic-induced apoptosis in the developing brain (Fig. 8a). We found that ERK phosphorylation was restored by hydrogen administration in the presence of sevoflurane (Fig. 8c,d: pERK1, one-way ANOVA, F = 29.69, *P* < 0.0001; pERK2, one-way ANOVA, F = 22.86, *P* < 0.0001), concomitant with an attenuation of apoptosis (Fig. 8b: PARP, one-way ANOVA, F = 69.09, *P* < 0.0001)). Conversely, hydrogen did not alleviate SL327-induced apoptosis (Fig. 8e). Hydrogen did not alter ERK phosphorylation in vehicle-treated mice (Fig. 8f). Thus, the effects of SL327 were independent of oxidative stress. Taken together, these results indicate that decrease of ERK phosphorylation is downstream of ROS production in sevoflurane-induced apoptosis.

## Discussion

In the current study, we observed that ERK phosphorylation was decreased in mice brain exposed to sevoflurane at P6, as reported previously^12^. Effects of decreased ERK phosphorylation was estimated by using SL327 and we found that transient suppression of ERK phosphorylation at P6 reproduced many sevoflurane-induced deleterious effects. Importantly, increased apoptosis caused by SL327 was apparently occluded in mice exposed to sevoflurane (Fig. 6a), suggesting that decreased ERK phosphorylation contributes to a great extent to the mechanism underlying anesthetic-induced toxicity in the developing brain. Furthermore, we observed that restoring ERK phosphorylation by hydrogen attenuated apoptosis caused by sevoflurane. Consistent with our result, some laboratories have reported that restoring ERK phosphorylation attenuated anesthetic-induced apoptosis in the developing brain^12^,^20^. Straiko *et al.* (2009) reported that anesthetic-induced neuro-apoptosis in the developing brain was attenuated by lithium’s alleviation of ERK suppression^12^. Wang *et al.* also reported that N-stearoyl-L-tyrosine attenuated anesthetic-induced neuro-apoptosis via upregulation of the ERK pathway in the developing brain^20^.

Taken together, our results strongly suggest that ERK suppression is critically involved in the mechanism underlying anesthetic-induced toxicity in the developing brain, although we could not exclude the possibility of existence of ERK-independent mechanism. Consistent with this notion, the effects of SL327 at P6 was much similar to those of sevoflurane. Anatomical pattern of apoptosis by SL327 and the cell type undergoing apoptosis were similar in mice administered SL327 and sevoflurane. Behavioral deficits properties caused by SL327 were also similar to those by sevoflurane. SL327 administration at P6 caused profound and long-lasting deleterious effects on behaviors. In the current study, we observed that mice exposed to SL327 exhibited hyperactivity in a familiar environment compared to control mice, similar to mice exposed to sevoflurane at P6. Thus, altered ERK phosphorylation level in the developing brain may result in attention deficit hyperactivity disorder, one of the most prevalent childhood-onset psychiatric disorders, as adult. In addition, mice exposed to SL327 showed depression-like behavior in the forced swim and tail suspension tests, similar to mice exposed to sevoflurane at P6. We previously reported that administration of SL327 at P6 also caused other behavioral deficits, including learning impairments and social deficits^13^, which resembled those induced by sevoflurane^3^. These results indicated that ERK has critically important roles in the brain development during the neonatal stage in mice.

The reduced levels of ERK phosphorylation were not proportional to the levels of cleaved PARP in our results. The level of ERK phosphorylation after sevoflurane administration at P6 (~30% of control level) was higher than the levels after SL327 administration (~5% of control level), although the apoptosis levels induced by these compounds were similar (Fig. 1). One possibility is that there may be a threshold for ERK phosphorylation levels to induce apoptosis, although the precise mechanism is unknown. Another possibility is that effects of sevoflurane involve other mechanisms independent of the ERK pathway.

Our results indicated that sevoflurane decreased ERK phosphorylation through the production of ROS, although how ROS accumulation results in decreased ERK phosphorylation in the developing brain is still unknown. It is also unclear how decreased ERK phosphorylation leads in turn to apoptosis. It might be possible that decreased ERK phosphorylation results in actin cytoskeleton disorganization and impaired dendritic branching since ERK plays important roles in actin cytoskeleton organization^21^ and dendritic branching^22^. Interestingly, it was reported that general anesthetics cause apoptosis in part by actin cytoskeleton disorganization and impaired dendritic branching^23^. Further study will be needed to understand the precise mechanism.

One might doubt that anesthetic-induced apoptosis might be due to poor respiratory care during anesthesia in small animals. In this connection, it should be noted that SL327 does not have anesthetic effects such as hypnosis and sedation. In addition, SL327 did not significantly alter pH, arterial partial oxygen, or carbon dioxide pressure (Table 1), indicating that the apoptosis caused by SL327 was not attributable to an indirect effect such as hypoxia. Thus, admitting that suppression of ERK phosphorylation is critically involved in the mechanism underlying anesthetic-induced toxicity in the developing brain, it is not likely that these anesthetic-induced apoptosis may be entirely attributable to disturbance in physiologic function (e.g., hypoxia or hypercarbia).

The deleterious effects of SL327 were age-dependent, similar to anesthetic-induced apoptosis in the developing brain^1^,^3^,^24^,^25^. One possibility is that mice may be more susceptible to a reduction in ERK phosphorylation at P6 than at later periods (Figs 1, 2, 3). In addition, basal ERK phosphorylation level may be more susceptible to sevoflurane exposure at P6 compared to later periods, since the reduction of ERK phosphorylation by sevoflurane at P6 appears to be larger than the reductions at P14 and P21.

Although the underlying mechanism is unclear, there seems to exist a distinct time-window, during which ERK activation plays important roles in the normal development of neural functions. This time-window might closely correlate with the “critical period”, during which animals display an elevated sensitivity to certain environmental stimuli^26^. During the critical period, neural circuits may be refined by experiences, and particular experiences can have profound and long-lasting effects on behavior^26^. Increasing evidence suggests that any interruption of neuronal activity during the critical period can contribute to abnormal phenotypes (e.g., autism spectrum disorder (ASD)), although the pathogenic mechanisms are largely unknown^27^. Further study will be needed to reveal the relationship between anesthetic-induced toxicity in the developing brain and the critical period.

In conclusion, our results strongly suggest that suppression of ERK phosphorylation through oxidative stress would be critically involved in the mechanism underlying anesthetic-induced toxicity in the developing brain. It may be important to keep ERK phosphorylation during anesthesia to prevent any deleterious effects in the developing brain. Furthermore, our result suggest that various neurodevelopmental disorders may be generally linked to defects in ERK signaling in the developing brain.

## Materials and Methods

### Animals

All experiments were conducted according to the institutional ethical guidelines for animal experiments of the National Defense Medical College (Tokorozawa, Japan) and Tokyo Metropolitan Hospital and Institute of Gerontology (Tokyo, Japan). All experimental protocols were approved by the Ethics Committee for Animal Experimentation at the National Defense Medical College or the Ethics Committee for Animal Experimentation at the Tokyo Metropolitan Hospital and Institute of Gerontology. Adult C57BL/6 mice were housed three or four per standard shoebox breeding cage (approx. size of 170 mm x 290 mm, 150 mm high) with autoclaved wood-chip bedding materials. Mice were maintained on a 12-h light-dark cycle (lights on from 07:00 to 19:00) with room temperature at 22 ± 1 °C with 46 ± 2% humidity. Mice had ad libitum access to water and food.

### Anesthesia treatment

Sevoflurane anesthesia was performed as previously described^28^. Briefly, pups at P6 were placed in a humid chamber immediately after their removal from the maternal cage. Sevoflurane (2%) was administered with 30% oxygen as the carrier gas. Control mice were exposed to 30% oxygen. Total gas flow was 2 L/min.

### MEK inhibitor administration

ERK isoforms, ERK1 and ERK2, are activated through the phosphorylation of tyrosine and threonine residues by the upstream kinase MEK. ERK phosphorylation is suppressed by the MEK inhibitor, SL327 (ENZO, Farmingdale, NY), which penetrates the blood-brain barrier^13^. SL327 was dissolved in dimethyl sulfoxide (DMSO; final concentration: 25 μg/μl). SL327 was administered intraperitoneally in a single dose at a concentration of 50 mg/kg. Therefore, if the body weight of the subject mouse was 5 g, then 10 μl of the SL327 solution was injected. Control animals were administered an injection of the same volume of DMSO.

### Hydrogen treatment

Hydrogen gas (1.3%) was supplied as described previously^19^. Briefly, when sevoflurane evaporated, hydrogen gas was supplied as part of a pre-mixed carrier gas (30% O_2_ + H_2_), which was produced by the manufacturer (Saisan Co., Saitama, Japan). Total gas flow was 2 L/min.

### Western blot analysis

Western blot analysis of forebrain protein lysate was performed as previously described^15^. Primary antibodies included anti-cleaved PARP (#9544, rabbit polyclonal; Cell Signaling Technology, Beverly, MA), anti-ERK1/2 (#9102, rabbit polyclonal, Cell Signaling Technology), anti-phospho ERK1/2 (#9101, rabbit polyclonal, Cell Signaling), and anti-β-actin (A5441, mouse monoclonal, Sigma, St. Louis, MO). Secondary antibodies included horseradish peroxidase (HRP)-linked anti-rabbit IgG (#7074, goat polyclonal, Cell Signaling Technology) and HRP-linked anti-mouse IgG (#7076, horse polyclonal, Cell Signaling Technology).

### Immunohistochemistry

Immunohistochemistry was performed as previously described^19^. Briefly, paraffin sections (5 μm thick) were de-paraffinized and immersed in an unmasking solution (Vector H3300; Vector Laboratories, Burlingame, CA) for antigen retrieval, and heated in an autoclave (121 °C) for 5 min. The sections were then incubated in a blocking reagent (Dako, Glostrup, Denmark) for 30 min to reduce background staining. The sections were then incubated with primary antibodies overnight in a humidified chamber at 4 °C. The primary antibodies included anti-active caspase-3 (#9661, rabbit polyclonal, Cell Signaling Technology), anti-NeuN (MAB377, mouse monoclonal, Millipore, Billerica, MA), anti-GFAP (G3893, mouse monoclonal, Sigma-Aldrich, St. Louis, MO), and anti-CNPase (C5922, mouse monoclonal, Sigma-Aldrich).

For chromogenic staining, immunoreactivity was detected using peroxidase-conjugated secondary antibody (Dako EnVision+ system; Dako) and 3,3-diaminobenzine-tetrachloride (DAB, Vector Laboratories). The sections were counter-stained with hematoxylin. For fluorescent staining, immunoreactivity was detected using Alexa-Fluor 488-conjugated goat anti-mouse IgG secondary antibody (Molecular Probes, Eugene, OR) for primary antibodies derived from mouse. For primary antibody derived from rabbit, Alexa-Fluor 546-conjugated goat anti-rabbit IgG secondary antibody (Molecular Probes) was used. Sections were examined using a Nikon fluorescence microscope system (Nikon, Tokyo, Japan) with an electron-multiplying (EM) CCD digital camera (ImagEM, Hamamatsu Photonics, Hamamatsu, Japan). Immunostaining analysis was performed by an investigator blinded to the treatment conditions. Samples from at least 5 mice per experimental condition were examined.

### Behavioral studies

Behavioral studies were performed at 10 weeks of age, as described previously^13^. The subject mice used in the behavioral studies were age-matched male littermates. Each experimental observation was performed by the same experimenter, who was blinded to the treatment conditions.

### Home-cage activity test

The home-cage activity test assessed spontaneous locomotor activity, and was performed as described previously^15^. Briefly, mice were individually housed in cages with bedding, food, and water. Activity was assessed by the total distance traveled using an 8-channel Activity Monitoring System (O’Hara & Co., Ltd., Tokyo, Japan).

### Forced swim test

The forced swim test, also known as the behavioral despair test, evaluates depression-like behavior in rodents, and was performed as described previously^29^. Briefly, mice were individually placed in a cylinder (25 cm diameter, 46 cm deep) filled with two-thirds with water (25 ± 1 °C) for 6 minutes; the mobility of the subject animal was measured. Immobility time or, “floating behavior” (e.g., the lack of movement beyond those movements necessary to maintain balance, with the subject’s head above water) was used as a parameter to assess ‘hopelessness’, and therefore, depression-like behavior. Subject mice could not escape from the cylinder, and their feet could not touch the bottom. After each swim, mice were lightly towel-dried and introduced back into their home-cage. The water in the cylinder was changed between each subject.

### Tail suspension test

The tail suspension test, another assay of depression-like behavior, was performed as described previously^17^, with minor modifications. Briefly, a mouse was suspended from the edge of a desk by attaching its tail with adhesive tape. The adhesive tape was placed approximately 5–10 mm from the tip of the tail. The suspended animal was 600 mm away from the floor. Total duration of immobility (e.g., lack of movement of paws, with the head pointed downward) was measured for 6 minutes.

### Arterial blood gas analysis

Arterial blood sampling from the left cardiac ventricle was performed as previously described^13^. Briefly, after anesthesia, arterial blood was obtained from the left cardiac ventricle. Samples were analyzed using a blood gas analyzer (ABL800; Radiometer, Copenhagen, Denmark).

### Statistical analysis

Statistical analysis was performed using GraphPad Prism 6.0 (GraphPad Software Inc, La Jolla, CA). Comparisons of the group means were performed using a Student’s t-test, a one-way analysis of variance (ANOVA), or a two-way ANOVA followed by the Bonferroni post hoc test. *P* values of less than 0.05 were considered statistically significant. Data are presented as mean ± SEM.

## Additional Information

**How to cite this article**: Yufune, S. *et al.* Suppression of ERK phosphorylation through oxidative stress is involved in the mechanism underlying sevoflurane-induced toxicity in the developing brain. *Sci. Rep.* **6**, 21859; doi: 10.1038/srep21859 (2016).

## Figures and Tables

**Table 1:** Arterial blood gas analysis.

	0 h	6 h (Vehicle)	6 h (Sevo)	6 h (SL327)
pH	7.47 ± 0.03	7.27 ± 0.04	7.31 ± 0.03	7.28 ± 0.09
PaO2 (mmHg)	132.2 ± 2.5	130.3 ± 2.8	132.3 ± 2.2	133.3 ± 3.2
PaCO2 (mmHg)	37.5 ± 4.7	43.9 ± 5.4	45.4 ± 3.6	41.4 ± 7.2

No significant differences in pH, PaO_2,_ and PaCO_2_ were observed between the various groups (one way ANOVA, *P* values > 0.05). n = 6 mice for each group. PaO_2_: partial pressure of arterial oxygen, PaCO_2_: partial pressure of arterial carbon dioxide. Values are presented as mean ± SEM.
